# Algorithm-Based Meta-Analysis Reveals the Mechanistic Interaction of the Tumor Suppressor LIMD1 With Non-Small-Cell Lung Carcinoma

**DOI:** 10.3389/fonc.2021.632638

**Published:** 2021-03-31

**Authors:** Ling Wang, Ayrianna Sparks-Wallace, Jared L. Casteel, Mary E. A. Howell, Shunbin Ning

**Affiliations:** ^1^ Department of Internal Medicine, Quillen College of Medicine, East Tennessee State University, Johnson City, TN, United States; ^2^ Center of Excellence for Inflammation, Infectious Diseases and Immunity, Quillen College of Medicine, East Tennessee State University, Johnson City, TN, United States

**Keywords:** LIMD1, lung cancer, algorithm analysis, LUAD, LUSC

## Abstract

Non-small-cell lung carcinoma (NSCLC) is the major type of lung cancer, which is among the leading causes of cancer-related deaths worldwide. LIMD1 was previously identified as a tumor suppressor in lung cancer, but their detailed interaction in this setting remains unclear. In this study, we have carried out multiple genome-wide bioinformatic analyses for a comprehensive understanding of LIMD1 in NSCLC, using various online algorithm platforms that have been built for mega databases derived from both clinical and cell line samples. Our results indicate that LIMD1 expression level is significantly downregulated at both mRNA and protein levels in both lung adenocarcinoma (LUAD) and lung squamous cell carcinoma (LUSC), with a considerable contribution from its promoter methylation rather than its gene mutations. The *Limd1* gene undergoes mutation only at a low rate in NSCLC (0.712%). We have further identified LIMD1-associated molecular signatures in NSCLC, including its natural antisense long non-coding RNA LIMD1-AS1 and a pool of membrane trafficking regulators. We have also identified a subgroup of tumor-infiltrating lymphocytes, especially neutrophils, whose tumor infiltration levels significantly correlate with LIMD1 level in both LUAD and LUSC. However, a significant correlation of LIMD1 with a subset of immune regulatory molecules, such as IL6R and TAP1, was only found in LUAD. Regarding the clinical outcomes, LIMD1 expression level only significantly correlates with the survival of LUAD (p<0.01) but not with that of LUSC (p>0.1) patients. These findings indicate that LIMD1 plays a survival role in LUAD patients at least by acting as an immune regulatory protein. To further understand the mechanisms underlying the tumor-suppressing function of LIMD1 in NSCLC, we show that LIMD1 downregulation remarkably correlates with the deregulation of multiple pathways that play decisive roles in the oncogenesis of NSCLC, especially those mediated by EGFR, KRAS, PIK3CA, Keap1, and p63, in both LUAD and LUSC, and those mediated by p53 and CDKN2A only in LUAD. This study has disclosed that LIMD1 can serve as a survival prognostic marker for LUAD patients and provides mechanistic insights into the interaction of LIMD1 with NSCLC, which provide valuable information for clinical applications.

## Introduction

Lung cancer is among the leading causes of cancer-related deaths in the world. There are nearly 230 thousand of new lung cancer patients and more than 135 thousand of deaths in 2020 in the USA (American Cancer Society). The 5-year relative survival rate of lung cancer from 1995 to 2001 was 15.7%. Non-small-cell lung carcinoma (NSCLC) is the major histological type of lung cancer that contributes to approximately 85% of all lung cancers. Like small-cell lung carcinoma (SCLC), NSCLC is originated from epithelial cells, and includes two main subtypes: lung adenocarcinoma (LUAD) and lung squamous cell carcinoma (LUSC). NSCLC patients are relatively insensitive to chemotherapy and radiation therapy compared with SCLC patients, and innovative therapeutic strategies are desired.

The adaptor protein LIMD1 is a member of the ZYXIN family (1). In humans, the *Limd1* gene is located at chromosome 3p21.3 (2), a region that undergoes frequent loss of heterozygosity in many solid tumors including both small and non-small cell lung cancers (2–7). LIMD1 possesses either pro-oncogenic or anti-oncogenic properties in different contexts. It was originally identified as a tumor suppressor in lung cancer (2, 8), and also plays a tumor-suppressing role in gastric cancer (9). It has been shown to regulate metastasis in breast, lung, and gastric cancers (2, 9, 10). In contrast, our recent studies have shown that LIMD1 plays a pro-oncogenic role in hematological malignancies in association with the oncogenic transcription factor interferon regulatory factor 4 (IRF4) (11, 12).

A few mechanisms underneath LIMD1-mediated suppressing functions in cancer have been disclosed. LIMD1 can interact with context-specific factors to execute its functions. For example, LIMD1 interacts with the tumor suppressor Rb to inhibit E2F-mediated gene transcription in the nucleus in AML (2), with VHL, PHD, and RHOBTB3 to repress HIF1α activity by promoting its ubiquitination-mediated degradation in response to cancer hypoxia (5, 13), and with LATS and WW45 to negatively regulate the anti-oncogenic Hippo signaling (14). Thus, its interacting partners play determinant roles in its functional consequences.

To gain a comprehensive understanding of the association of LIMD1 with the development of NSCLC, in this study, we have employed various online algorithms to conduct secondary analyses of available datasets. We show that LIMD1 expression is significantly downregulated in NSCLC, and have identified LIMD1-associated tumor-infiltrating immune cells (TILs), and molecular and immune signatures in this setting. More importantly, we found that LIMD1 significantly correlates with the survival of LUAD but not that of LUSC patients.

## Methods

We employed different online algorithms for metadata analysis, including Oncomine, Genotype-Tissue Expression (GTEx), Gene Expression Atlas, ProteinAtlas, ProteomicsDB, Tumor Immune Estimation Resource (TIMER v2) (15, 16), Gene Expression Profiling Interactive Analysis (GEPIA v2) (17), Tumor-Immune System Interactions Database (TISIDB) (18), UALCAN (19), COSMIC, Tumor Fusion Gene Data Portal (TumorFusions) (20), FusionGDB (21), ChimerDB v4 that integrates several different fusion portals such as STARFusion, TCGA-FAWG, and FusionScan (22), cBioportal (23, 24), DriverDBv3 (25), Kaplan Meier Plotter (KMPlot) (26), muTarget (27), SMART (28), Lung cancer explorer (LCE), and ENSEMBL, for mRNA and protein expression, correlation, gene mutation, promoter methylation, fusion, tumor-immune interaction, and survival analyses. All portals include the Cancer Genome Atlas (TCGA) datasets, in addition to other unique datasets obtained from patients and cell lines. BioGRID (29, 30), GeneMANIA (31, 32), STRING, Uniprot, and KEGG, and PhosphoSitePlus portals were applied for post-translational modifications, signaling pathway, and protein-protein and functional interaction analyses.

All analyses were carried out using the default settings of the corresponding algorithms if not otherwise indicated, with the detailed dataset information and guidelines available on each algorithm portal. p<0.05 is considered statistically significant and >0.05 is non-significant (n.s), and p<0.01 is considered statistically very significant.

## Results

### Tissue- and Cell-Specific Expression of LIMD1

**Figure 1:**
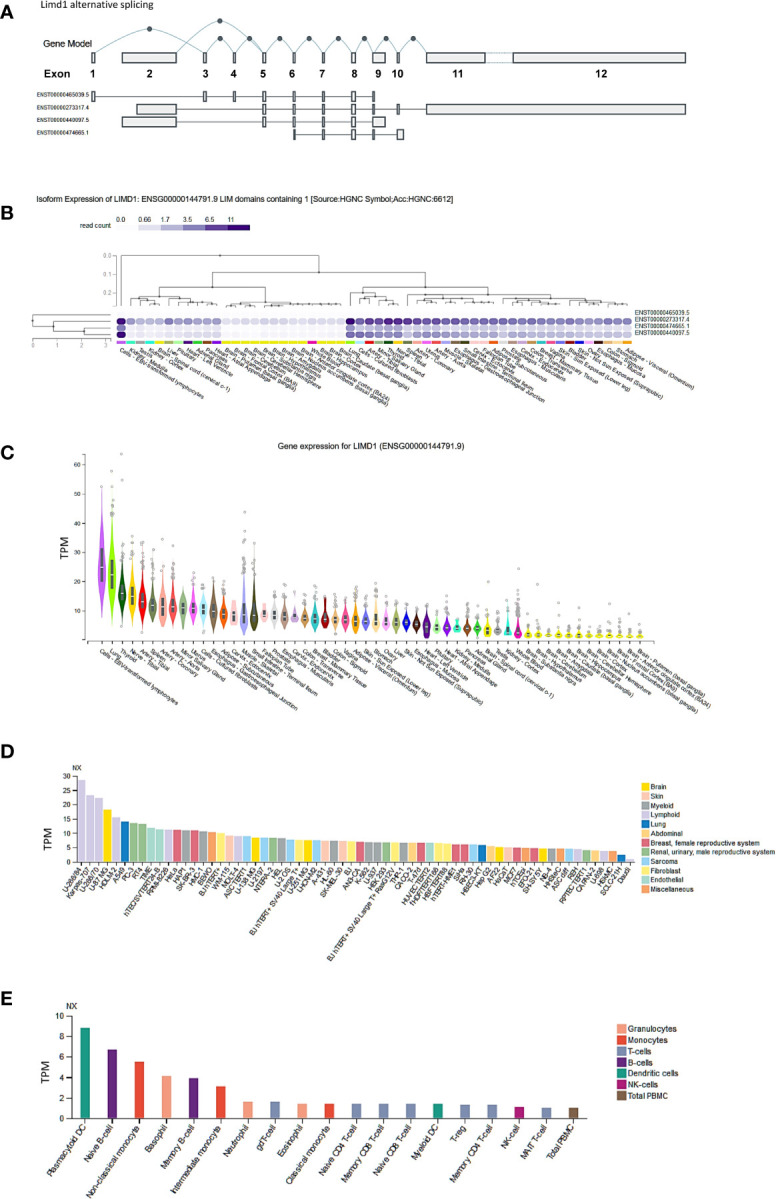
Tissue- and cell-specific expression of LIMD1. **(A)** Four splicing variants of *Limd1* primary transcript, two of which encode proteins (676 aa for the dominant transcript ENST00000273317.4 and 620 aa for ENST00000440097.5). **(B, C)** Tissue-specific expression of *Limd1* splicing variants and gene. **(D)** Cell-specific expression of *Limd1* transcript. **(E)** Cell-specific expression of *Limd1* transcript in the blood. **(A–C)** are the results from the GTEx portal, and **(D, E)** from the ProteinAtlas portal. TPM, transcripts per million; NX, denoted normalized expression.

To understand the role of LIMD1, we first evaluated its tissue- and cell-specific expression patterns in humans, in GTEx, Gene Expression Atlas, and ProteinAtlas portals, which include datasets obtained from human protein atlas (HPA), functional annotation of the mammalian genome (FANTOM v5), and GTEx projects. The *Limd1* gene produces four alternatively spliced variants (**Figure 1A**). The ENSEMBL portal shows that two of these four splice variants encode proteins, with the size of 676 aa for the dominant transcript ENST00000273317.4 and 620 aa for ENST00000440097.5. Analysis in the GTEx portal indicates that three of the four splice variants are widely expressed in various tissues and cell lines (**Figure 1B**), with the highest levels in lung and Epstein-Barr virus (EBV)-transformed lymphoblastic cell lines (LCLs) (**Figures 1B, C**). Analysis in ProteomicsDB shows that the encoded LIMD1 protein displays a tissue-specific differential expression pattern similar to that of *Limd1* transcription (data not shown). Consistent with these results, our recent studies have shown that LIMD1 is induced by NFκB and IRF4 downstream of the signaling pathway triggered by the EBV principal oncoprotein LMP1 in EBV-transformed LCLs, suggesting that LIMD1 could serve as an oncogenic biomarker in association with NFκB and IRF4 in certain hematological malignancies (11, 12).

Regarding cell-specific expression, analysis in the ProteinAtlas portal shows that the transcript abundance of LIMD1 is mainly enriched in the cells originated from lymphoid, brain, and lung (**Figure 1D**). In the blood, LIMD1 is mainly expressed in plasmacytoid dendritic cells, followed by B cells, basophils, and monocytes (**Figure 1E**).

### LIMD1 Is Downregulated in NSCLC

**Figure 2:**
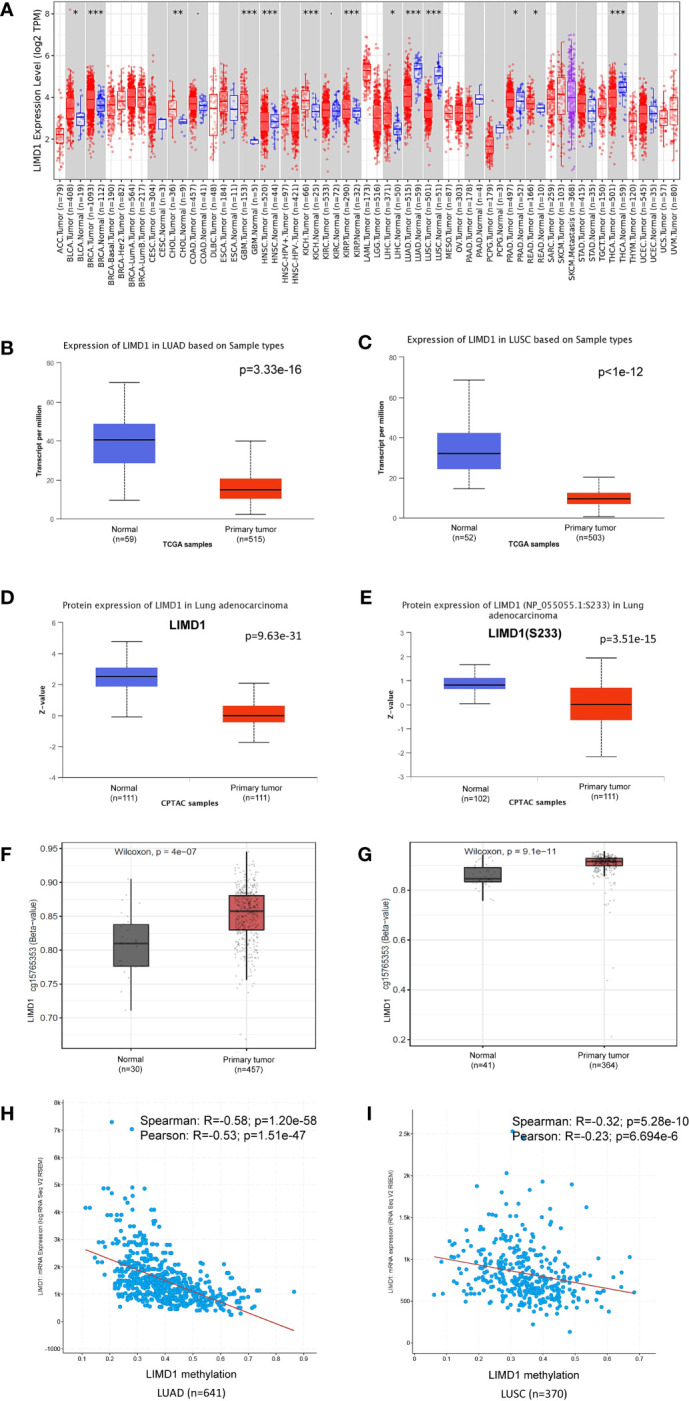
LIMD1 is downregulated in NSCLC. **(A)** Deregulation of LIMD1 at the transcriptional level in various cancers (*: p < 0.05; **: p<0.01; ***: p<0.001). Blue: Normal tissues; Red: Tumor tissues. Pink: Tumor tissues without normal tissue controls; Purple: Metastasis tumor tissues. ACC: Adrenocortical carcinoma; BLCA: Bladder Urothelial Carcinoma; BRCA: Breast invasive carcinoma; CESC: Cervical squamous cell carcinoma and endocervical adenocarcinoma; CHOL: Cholangiocarcinoma; COAD: Colon adenocarcinoma; DLBC: Diffuse large B-cell lymphoma; ESCA: Esophageal carcinoma; GBM: Glioblastoma multiforme; HNSC: Head and Neck squamous cell carcinoma; KICH: Kidney chromophobe; KIRC: Kidney renal clear cell carcinoma; KIRP: Kidney renal papillary cell carcinoma; LAML: Acute myeloid leukemia; LGG: Brain lower-grade glioma; LIHC: Liver hepatocellular carcinoma; LUAD: Lung adenocarcinoma; LUSC: Lung squamous cell carcinoma; MESO: Mesothelioma; OV: Ovarian serous cystadenocarcinoma; PAAD: Pancreatic adenocarcinoma; PCPG: Pheochromocytoma and paraganglioma; PRAD: Prostate adenocarcinoma; READ: Rectum adenocarcinoma; SARC: Sarcoma; SKCM: Skin cutaneous melanoma; STAD: Stomach adenocarcinoma; TGCT: Testicular germ cell tumors; THCA: Thyroid carcinoma; THYM: Thymoma; UCEC: Uterine corpus endometrial carcinoma; UCS: Uterine carcinosarcoma; UVM: Uveal melanoma. **(B, C)** LIMD1 transcription is downregulated in LUAD and LUSC, respectively. **(D, E)** LIMD1 protein level and S233 phosphorylation are downregulated in LUAD. **(F, G)** LIMD1 promoter is significantly methylated in both LUAD and LUSC. **(H, I)** LIMD1 gene transcription negatively correlates with its promoter methylation in LUAD and LUSC, respectively. **(A)** is the results from the TIMER2 portal, **(B, G)** from UALCAN, and **(H, I)** from cBioportal.

We next analyzed LIMD1 mRNA abundance in different human cancers, using Oncomine, TIMER2.0, GEPIA2, UALCAN, and DriverDBv3. Results from these different portals show that LIMD1 transcript is significantly downregulated in NSCLC (LUAD and LUSC), uterine corpus endometrial carcinoma (UCEC), and uterine carcinosarcoma (UCS), and upregulated in lymphoid neoplasm diffuse large B-cell lymphoma (DLBC), breast cancer (BRCA), glioblastoma multiforme (GBM), acute myeloid leukemia (LAML), and brain lower-grade glioma (LGG) (**Figures 2A-C**; **Figure 5C**; **Supplementary. Figures 1**, **2**). In agreement with these results, LIMD1 deregulation was previously found in lung cancer, non-Hodgkin lymphomas, gastric and colorectal carcinomas, and breast cancer (8, 33–35). The portal Oncomine comprises different datasets with lung cancer clinical or cell line samples, and only those within our statistical power and appropriate controls were selected for analysis (**Figure 2**). Consistently, LIMD1 protein and S233 phosphorylation levels are downregulated in LUAD and LUSC, as analyzed in UALCAN (**Figures 2D, E**). In fact, S233 phosphorylation of LIMD1 has been identified by high throughput profiling assays in many other cancers, including breast and ovarian cancers and pancreatic ductal adenocarcinoma, with or without stresses (36–38).

Promoter methylation analysis of TCGA lung cancer dataset in SMART portal indicates that the *Limd1* gene promoter is highly methylated in a region spanning nucleotides 45559963 to 45678725 in both LUAD and LUSC, exemplified by the probe cg15765353 that covers the two nucleotides 45559963-4 (p=4e-7 for LUAD and p=9.1e-11 for LUSC) (**Figures 2F, G**). Pearson and Spearman correlation analyses in cBioportal, which includes TCGA and other lung cancer datasets, show that the *Limd1* gene promoter methylation significantly correlates with its downregulation in both LUAD and LUSC cancers, with a greater correlation coefficient in LUAD (**Figures 2H, I**). Similar results were obtained in DriverDBv3 for TCGA lung cancer dataset (R=-0.361; p=3.16e-17 for LUAD vs R=-0.246; p=2.92e-8 for LUSC). These findings indicate that *Limd1* gene promoter methylation makes a significant contribution to the downregulation of LIMD1 expression in NSCLC.

**Figure 3:**
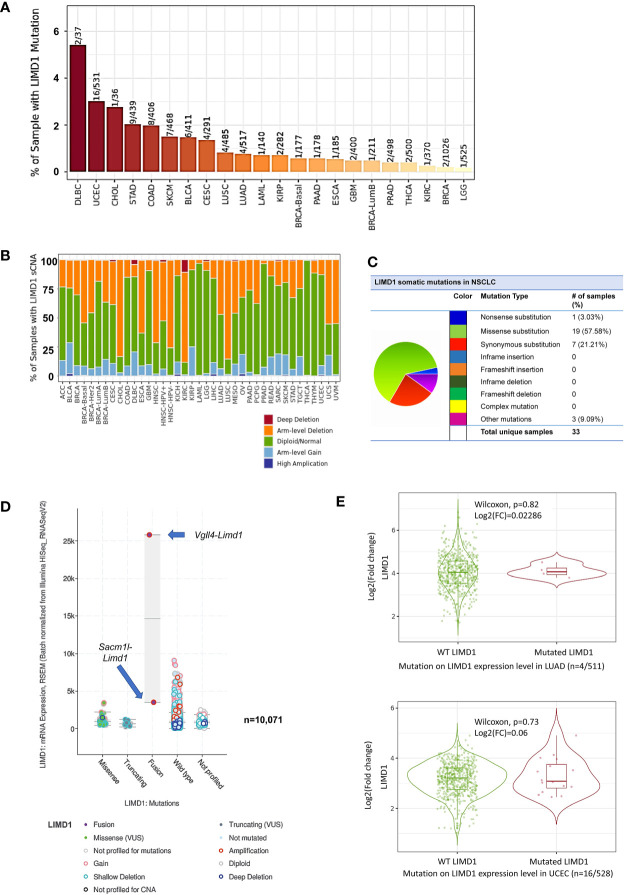
Profile of *Limd1* gene mutation in cancers. **(A)** Frequencies of *Limd1* gene mutation in various cancers. **(B)** Frequencies of *Limd1* somatic copy number alteration (sCNA). **(C)** Frequencies of *Limd1* somatic mutations (substitutions, deletions, and insertions) in NSCLC. **(D)** Effects of LIMD1 mutation on its expression in various cancers. **(E)** Representative results from LUAD and UCEC show that LIMD1 mutation has no significant effects on its expression. **(A, B, E)** are the results from TIMER2, **(C)** from COSMIC, and **(D)** from cBioportal. FC, Fold change.

Most, if not all, neoplasms generated in response to environmental carcinogens result from mutations that initiate cancerogenesis. In general, a given cancer requires 2–8 mutations to initiate (39). Mutation analysis of TCGA dataset in TIMER, cBioportal, and DriverDBv3 portals shows that the *Limd1* gene undergoes low rates of mutation in NSCLC (4/485 in LUSC and 4/517 in LUAD), but undergoes higher rates in DLBC (2/37), UCEC (16/531), and cholangiocarcinoma (CHOL, 1/36) (**Figure 3A**). Regarding lung cancer, in agreement with previous reports (4, 40), TCGA lung cancer cohort comprises high frequencies of *Limd1* arm-level deletion correlating with somatic copy number alteration (sCNA), with LUSC (>75%) being higher than LUAD (~50%) (**Figure 3B**). Further analysis in COSMIC portal shows that *Limd1* cDNA somatic mutations (substitutions, deletions, and insertions) were detected in 33 out of 226 lung cancer samples (14.6%), with most being missense substitution (57.58%) (**Figure 3C**). cBioportal analysis shows that *Limd1* has a rate of 0.712% of mutations in NSCLC patients (43/6040) (not shown). ChimerDB and TumorFusions portals indicate that, among these detected *Limd1* mutation types, *Limd1*-*Lars2* gene fusion occurs in both LUAD and LUAC tumors and also in STAD, with Tier 1 in-frame fusion in LUAD and Tier 2 out-of-frame fusion in LUSC (**Supplementary Figure 3**). cBioportal analysis shows that *Dhx30*-*Limd1* fusion occurs in PRAD, *Sacm1l*-*Limd1* fusion occurs in BRCA, and *Vgll4*-*Limd1* fusion occurs in BLCA, with *Vgll4*-*Limd1* fusion resulting in a remarkable increase of LIMD1 expression (**Figure 3D**). FusionGDB indicates that two other fusion genes, *Limd1-Dsp* and *Plec-Limd1*, exist in diseases other than cancers. Consistent results from different portals, including cBioportal and TIMER2, indicate that the overall *Limd1* mutation in different cancers has no significant effects on its expression (**Figures 3D, E**), although the analysis was not performed for some types of cancer in that their sample sizes with *Limd1* mutation are not powerful enough.

Together, these findings support the claim that the deregulation of LIMD1 expression in different cancers mainly results from epigenetic reprogramming rather than its mutation, with its downregulation in NSCLC being partially attributed to promoter methylation and arm-level deletion. In addition to these epigenetic mechanisms, miRNAs likely regulate LIMD1 expression in different cancer contexts, with miR-127-5p, miR-147a, and miR-130b-3p that potentially target LIMD1 in LUAD, as analyzed in DriverDBv3 (**Supplementary Table 1**).

### LIMD1 Differential Expression and Correlation Patterns in NSCLC

**Figure 4:**
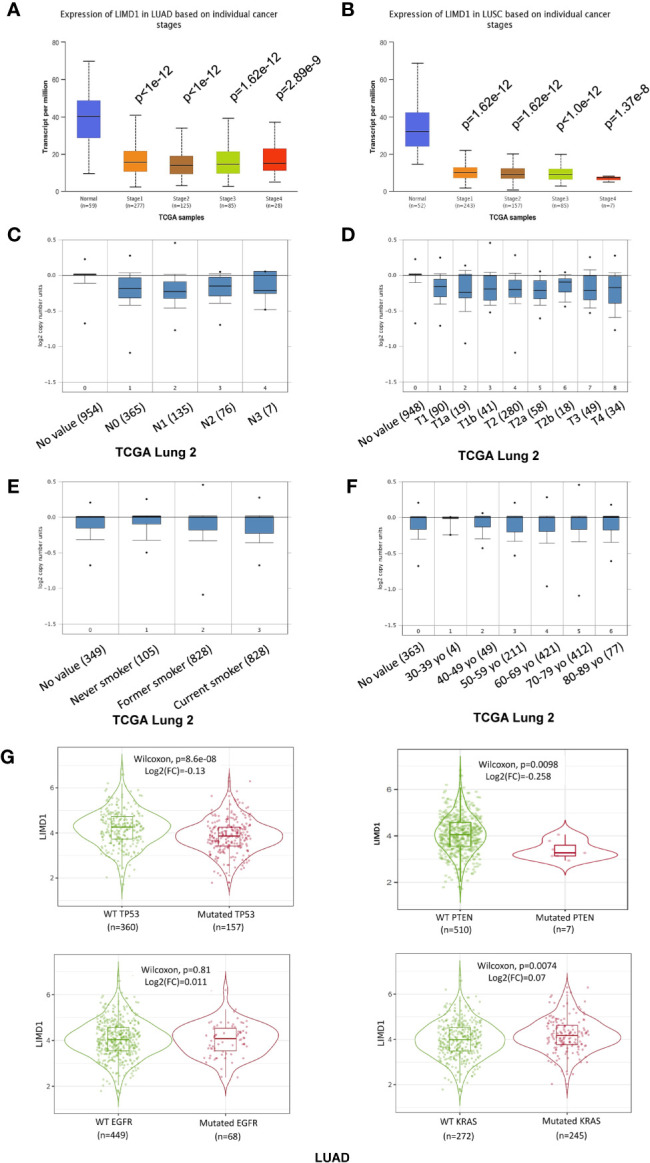
LIMD1 differential expression patterns in lung cancer stages and its regulation by gene mutation. **(A, B)** LIMD1 is downregulated at the early stages of NSCLC. **(C, D)** Clinical stages of lung cancer have no significant differences in LIMD1 expression. **(E)** Smoke status has no significant effects on LIMD1 expression. **(F)** Ages have no significant effects on LIMD1 expression. **(G)** Effects of mutation of key lung cancer genes on LIMD1 expression in LUAD. **(A, B)** are the results from UALCAN portal, **(C, F)** from Oncomine, and **(G)** from TIMER2.

We further analyzed TCGA lung cancer 2 dataset, which includes 1,537 lung cancer samples (420 blood and 1,117 tissue samples, comprising 370 LUAD, 357 LUSC, and 810 unclassified), in the Oncomine portal. Results show that LIMD1 downregulation occurs at the early stage of cancer development in both LUAD and LUSC, and there are no significant differences between every two stages (**Figures 4A, B**). However, smoke status, ages, gender, and race have no significant correlation with LIMD1 deregulation in NSCLC (**Figures 4A, F**; data not shown for gender and race). Similar results were obtained from other lung cancer datasets in Oncomine and also from the analysis in the UALCAN portal (not shown).

The genes encoding p53, KRAS, EGFR, ALK, PTEN, and PI3K are frequently mutated in lung cancers (**Supplementary Figure 4**). We thus assessed whether mutation of these key genes correlates with LIMD1 expression levels in the TIMER2 portal. Results show that mutation of p53 (p=8.6e-8; log2(FC)=-0.13) and PTEN (p=0.0098; log2(FC)=-0.258) genes correlates with LIMD1 downregulation in LUAD but not LUSC patients (**Figure 4G**; data for LUSC not shown). Similar results were obtained for p53 mutation in the UALCAN portal (p=7.535e-08 for LUAD and p=0.962 for LUSC).

In addition to these frequently mutated genes, we further analyzed whether mutations of any other genes may affect LIMD1 expression in NSCLC in the muTarget portal. Results show that, in a panel of mutated genes (**Supplementary Table 2**), OR4C15, RALGAPA1, SPAM1, TRPV3, and WDR17 genes in LUAD, and AMPD3 in LUSC are at the top rank whose mutation significantly results in LIMD1 downregulation (p<0.01; fold change>1.44) (**Supplementary Figure 5**).

**Figure 5:**
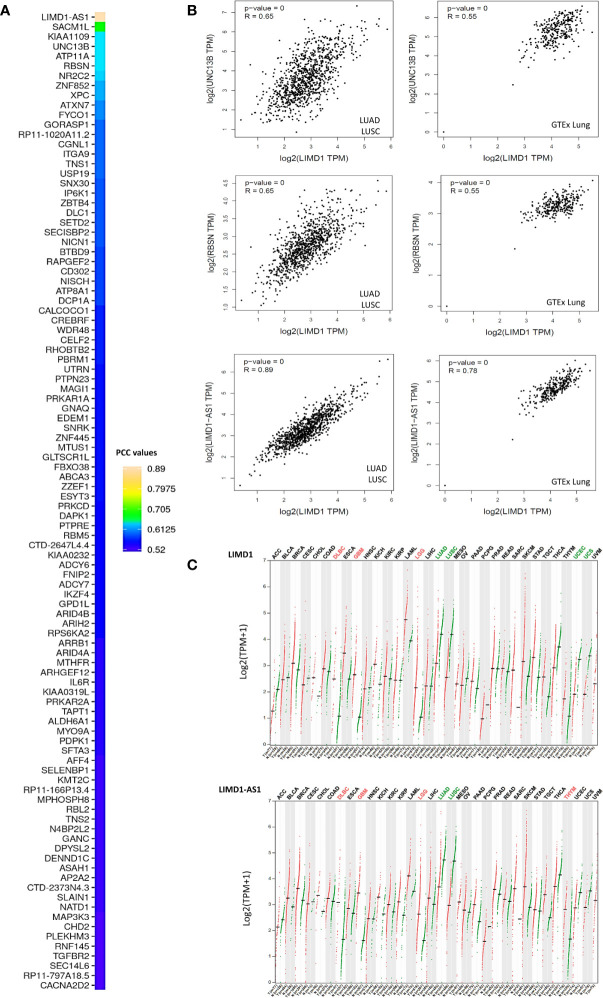
LIMD1-associated molecular signature in NSCLC. **(A)** Profiling genes coexpressed with LIMD1 in NSCLC in GEPIA2. Pearson correlation coefficient values (PCC) >0.5 are shown. **(B)** Validation of individual genes in association with LIMD1 transcription in GEPIA2. **(C)** Similar expression patterns of LIMD1 and LIMD1-AS1 across various cancer versus normal tissues, as analyzed in GEPIA2. Cancer types are labeled on the top of the graphs, with red fonts showing significant upregulation and green fonts showing significant downregulation. T, Tumor tissues (Red dots); N, Normal tissues (Green dots).

We next performed genome-wide association studies (GWAS) to profile LIMD1-associated molecular signatures in lung cancer, in Oncomine, GEPIA2, and UALCAN portals, using the Pearson correlation test. These analyses identified a panel of common genes that correlate with LIMD1 (PCC>0.5) at the mRNA level in both LUAD and LUSC, and representative results from the GEPIA2 portal are shown in **Figure 5A**. Among these common genes, representative ones include LIMD1-AS1, which encodes LIMD1 natural antisense (NAT) long non-coding RNA (lncRNA), and those encoding the membrane trafficking regulators RBSN, SACM1L, UNC13B, and ATP11A. We validated the correlation of LIMD1 with selected genes in both TCGA and GTEx lung cancer datasets, by one-to-one paired analysis in GEPIA2 and/or TIMER2 portals, and representative results for UNC13B, RBSN, and LIMD1-AS1 are shown in **Figure 5B**.

LIMD1-AS1 perfectly correlates with LIMD1 not only in lung cancer, but also in all other types of cancers in the TCGA dataset (**Figure 5C**). In support of this observation, LIMD1-AS1 was recently shown to stabilize LIMD1 mRNA in a hnRNP-U-mediated manner in lung cancer (41). RBSN belongs to the FYVE zinc finger family, members of which bind to the membrane lipid phosphatidylinositol-3-phosphate (PtdIns(3)P) with high specificity. SACM1L is a phosphoinositide phosphatase that catalyzes the hydrolysis of PtdIns(4)P. UNC13B plays a role in vesicle maturation during exocytosis, and ATP11A is the catalytic component of a P4-ATPase flippase complex that catalyzes the hydrolysis of ATP coupled to the transport of amino-phospholipids from the outer to the inner leaflet of various membranes. These intriguing findings imply that LIMD1 has an intimate association with membrane trafficking. In support of this role, the LIMD1 proteins have been reported to play a role in actin filament assembly and cytoskeletal organization (1, 42) we have collected a pool of preliminary data showing that LIMD1 is important for autophagosome biogenesis induced by oxidative stress in cancer cells (to be published).

Additionally, we identified LUAD- and LUSC-specific genes that correlate with LIMD1 at the transcriptional level in GEPIA2 and UALCAN portals (**Supplementary Table 3**).

### LIMD1 Expression Level Correlates With the Frequencies of Distinct Tumor-Infiltrating Lymphocytes in LUAD and LUSC

Tumor-infiltrating lymphocytes (TILs), an important component of the tumor microenvironment (TME), can recognize and kill cancer cells, and are an ideal candidate for cellular immunotherapy for various cancers (43–46). Especially in NSCLC, where the efficacy of immune checkpoint inhibitors (ICIs) is a concern since only a limited percentage of NSCLC patients have gotten promising prognoses (47, 48). Thus, the identification of novel biomarkers associated with TILs is of great importance in NSCLC clinical applications.

**Figure 6:**
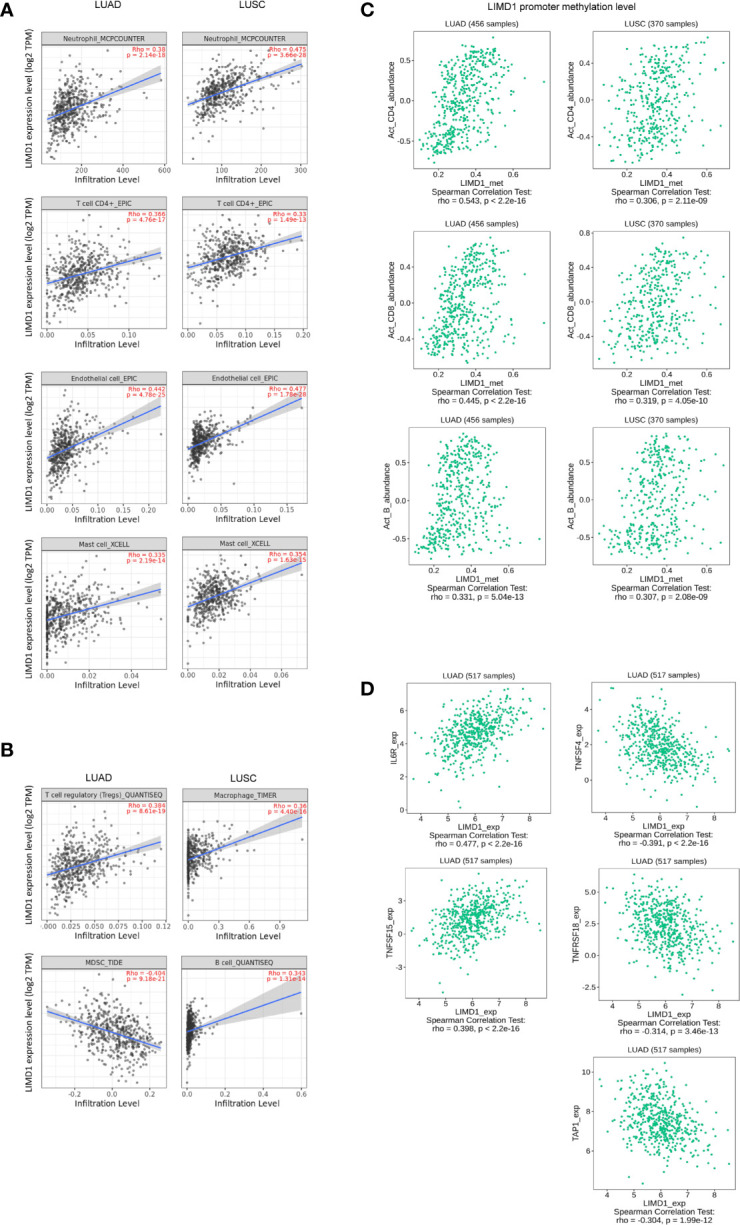
LIMD1 correlates with immune infiltration in NSCLC. **(A, B)** LIMD1 expression significantly correlates with a subgroup of immune infiltrating lymphocytes in LUAD and LUSC. **(C)** Methylation of the *Limd1* gene promoter significantly correlates with immune infiltration. **(D)** LIMD1 expression significantly correlates with a subset of immune regulatory molecules in LUAD. **(A, B)** are the results from TIMER2, and **(C, D)** from TISIDB.

We, therefore, sought to assess the correlation of LIMD1 expression with different subtypes of TILs in NSCLC, including CD4 T cells, CD8 T cells, B cells, NK cells, dendritic cells (DCs), macrophages, myeloid-derived suppressor cells (MDSCs), mast cells, neutrophil, eosinophils, cancer-associated fibroblasts, endothelial cells, common lymphoid and myeloid progenitors, and hematopoietic stem cells, using Spearman correlation test in TIMER2 portal. We set the cutoff values of the Spearman coefficient to R<-0.33 (negative correlation) or R>0.33 (positive correlation), and p<0.05 was considered significant. Within these cutoff criteria, we found that LIMD1 expression levels positively correlate with the infiltration of neutrophils, CD4+ T cells, endothelial cells, and mast cells in both LUAD and LUSC (**Figure 6A**). Of note, among all subtypes of TILs, neutrophils are the majority in both LUAD and LUSC (the infiltration level is peaked approximately at 100 in LUAD and LUSC) (**Figure 6A**). In agreement with this finding, neutrophils were reported to dominate the immune cell composition in NSCLC (49). Further, LIMD1 significantly correlates with tumor-infiltrating regulatory T cells (Treg) and negatively correlates with tumor-infiltrating MDSCs in LUAD, and also significantly correlates with tumor-infiltrating macrophages and B cells in LUSC (**Figure 6B**). These findings imply that these TILs may play plausible and unique roles in regulating anti-tumor immunity in LUAD and LUSC, although currently, they are not ideal pharmacological targets.

We further show that *Limd1* gene promoter methylation levels significantly correlate with the frequencies of activated tumor-infiltrating CD4+ and CD8+ T cells and B cells (R<-0.30 or R>0.30 and p<0.05), with greater correlation coefficients in LUAD compared with LUSC (**Figure 6C**).

We next evaluated LIMD1 in correlation with immune regulatory molecules (suppressors and stimulators) at their expression levels. Within the cutoff values of R<-0.30 or R>0.30 and p<0.05, we identified LIMD1 mRNA abundance significantly correlates with IL6R and TNFSF15, and negatively correlates with TNFSF4 and TNFSF18. LIMD1 also negatively correlates with the MHC molecular TAP1 (**Figure 6D**). However, no correlation between LIMD1 and any of these molecules in LUSC within the criteria (not shown).

### High LIMD1 Expression Levels Favor the Prognosis of LUAD but Not LUSC Patients

**Figure 7:**
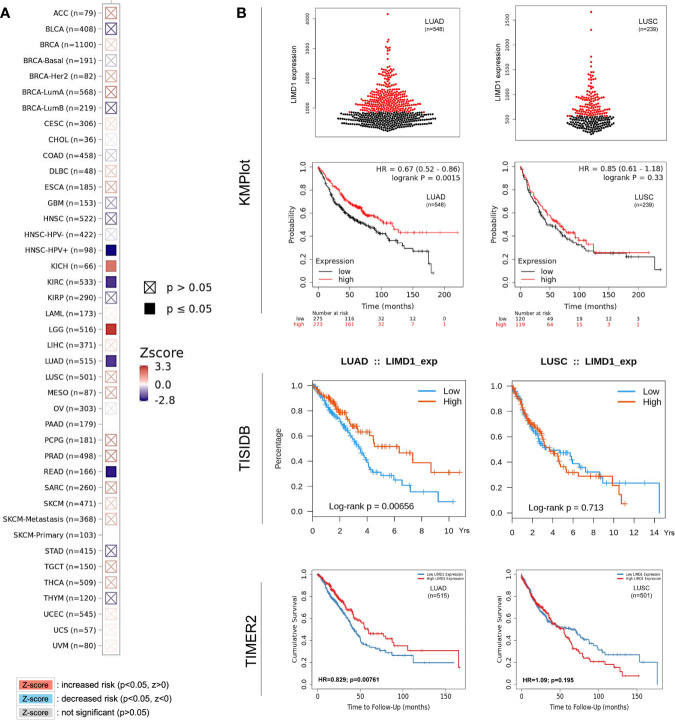
LIMD1 correlates with the overall survival of certain cancers. **(A)** LIMD1 expression level correlates with the overall survival (OS) of certain cancer patients (200 months). **(B)** LIMD1 expression level significantly correlates with OS of LUAD patients, but not with LUSC patients. **(A)** are results from TIMER2, and **(B)** from different portals showing the consistency. For KMPlot, the settings were: only JetSet best probe set, excluding outlier arrays, and univariate.

Regarding the clinical outcomes of LIMD1 deregulation in NSCLC, we assessed the prognosis values of LIMD1 deregulation in various cancers. To this end, the relation between LIMD1 expression levels and the overall survival (OS) rates of cancer patients was analyzed in different portals, including TIMER2, TISIDB, GEPIA2, and DriverDB portals with TCGA dataset, and Kaplan Meier plotter (KMPlot) portal with TCGA, EGA, and GEO datasets. We found that the abundance of LIMD1 is significantly associated with overall survival (OS) of HPV-positive HNSC, KICH, KIRC, LGG, LUAD, and READ, and representative results from TIMER2 are shown in **Figure 7A**.

Regarding the prognostic implications of LIMD1 in NSCLC patients, similar results were obtained from TIMER2, TISIDB, DriverDBv3, and KMPlot portals showing that the LIMD1 expression levels positively correlate with the overall survival (OS) of LUAD but not that of LUSC patients (HR=0.67, p=0.0015 in KMPlot; p=0.00656 in TISIDB; HR=0.829, p=0.00761 in TIMER2; and HR=0.616, p=0.00328 in DriverDBv3 for LUAD. HR=0.85, p=0.33 in KMPlot; p=0.713 in TISIDB; HR=1.09, p=0.195 in TIMER2; and HR=1.42, p=0.0109 in DriverDBv3 for LUSC) (**Figure 7B**; Results from DriverDBv3 not shown). These results indicate that LIMD1 can serve as a promising prognostic biomarker for LUAD (but not for LUSC). In line with our findings, LIMD1 was shown as a prognostic indicator for NSCLC, and its loss significantly worsened 5-year OV of the patients (4). These findings underline the correlation between LIMD1 expression and clinical outcomes in LUAD patients.

### Possible Mechanism Underneath Tumor-Suppressing Function of LIMD1 in NSCLC

**Figure 8:**
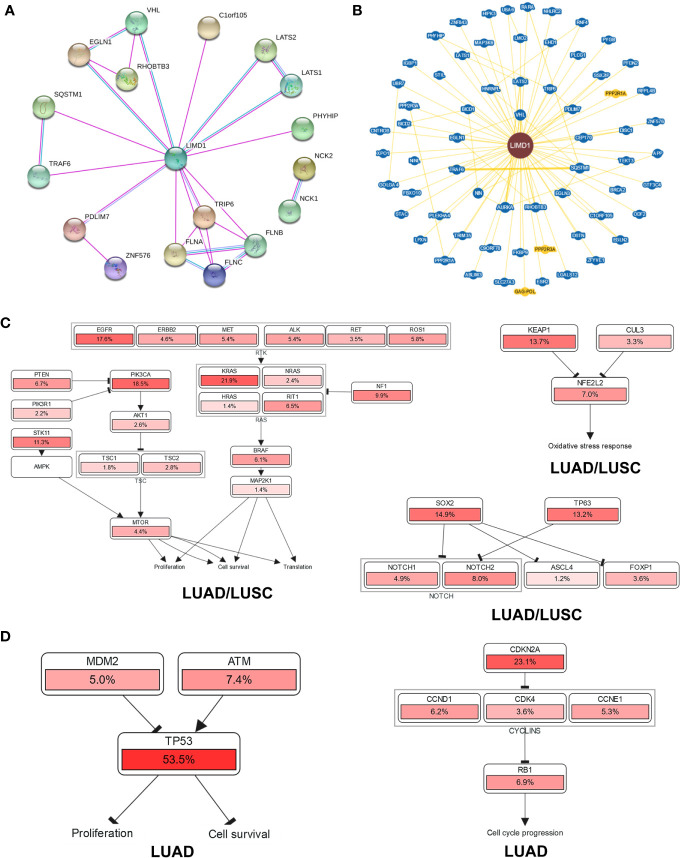
Genome-wide interaction and pathway association of LIMD1 in NSCLC. **(A)** Known LIMD1 protein physical interaction network, as analyzed in STRING databases. Co-expression, textmining, neighborhood, and gene fusion were excluded in the settings. Green line: from curated databases; Pink line: Experimentally validated; Purple line: Proteins with homology. **(B)** Physical interactors of LIMD1 protein as identified in BioGRID with various databases from both high and low throughput assays. Blue: Associated protein from the same organism; Yellow: Associated protein from a different organism (PPP2R1A and PPP2R3A are from mouse, and GAG-POL from HIV). **(C)** The pathways that are deregulated in correlation with LIMD1 in both LUAD and LUSC. **(D)** The unique pathways that are deregulated in correlation with LIMD1 in LUAD. The deregulation of individual genes is shown in red. The more frequently a given gene is deregulated, the deeper red is shown.

To explore the potential mechanisms responsible for LIMD1-mediated tumor suppression, we first analyzed genome-wide LIMD1 interaction network in STRING, BioGRID, and GeneMANIA. Results show that a spectrum of proteins physically interact with LIMD1 in humans, as discovered in both high and low throughput profiling assays (66 in BioGRID) (**Figures 8A, B**), in addition to RHOBTB3 (13), VHL (4, 5), TRAF6 (11, 50), SQSTM1 (p62) (51, 52), LATS1/2 (14, 53, 54), and FLNA/B/C (1), which are known to functionally or physically interact with LIMD1 (**Supplementary Table 4**). These LIMD1 interactors are involved in broad biological processes.

Finally, we analyzed the possible pathways with the involvement of LIMD1 in KEGG and Uniport (**Supplementary Figure 6**), and those associated with LIMD1 downregulation in NSCLC in cBioportal (**Figures 8C, D**). Results show that LIMD1 deregulation has a remarkable association with EGFR-RTK-KRAS and PI3K/mTOR pathways that regulate cell proliferation and survival, with Keap1-NRF2 pathway that serves as the master antioxidant defense mechanism, and with SOX2/p63 that inhibit NOTCH signaling (**Figure 8C**), in both LUAD and LUSC. More importantly, in support of its correlation with the survival of LUAD patients (**Figure 7**), LIMD1 downregulation greatly correlates with the deregulation of p53-mediated cell proliferation and survival, and with the deregulation of CDKN2A-Rb-mediated cell cycle progress, in LUAD but not in LUSC (**Figure 8D**).

## Discussion

In this algorithm-based study, we show that: 1) LIMD1 is significantly deregulated in several malignancies, including its downregulation in NSCLC (**Figure 2**); 2) LIMD1 promoter methylation, but not its mutations that occur at low rates in NSCLC, makes a significant contribution to its downregulation in NSCLC (**Figure 2**; **Figure 4**); 3) Downregulation of LIMD1 transcription occurs at early stages of NSCLC, and smoke status, ages, gender, and races of the patients do not correlate with its downregulation (**Figure 3**); 4) LIMD1 perfectly correlates with LIMD1-AS1 at the mRNA expression level in various cancers, including NSCLC, and also significantly correlates with a panel of membrane trafficking regulators in NSCLC (**Figure 5**). 5) LIMD1 significantly correlates (most positively) with several subtypes of TILs, with neutrophils being the majority of TILs, in both LUAD and LUSC. Correspondingly, LIMD1 expression correlates (positively or negatively) with a subgroup of immunomodulators (**Figure 6**). 6) More importantly, we found that LIMD1 deregulation is significantly associated with the prognosis of several malignancies, with its high levels favoring the prognosis of LUAD, but not LUSC (**Figure 7**). 7) Mechanistically, our analysis indicates that LIMD1 expression level correlates with the deregulation of various cellular functions, including cell proliferation and survival, oxidative stress, and cell cycle progress, which are mediated by EGFR, KRAS, PI3K, and Keap1, in both LUAD and LUSC, and specifically correlates with the deregulation of the pathways mediated by p53 and CDKN2A in LUAD (**Figure 8**).

Overexpression of LIMD1 was shown to be a hallmark of ABC type of DLBC (55). Our GEO high throughput profiling has revealed that LIMD1 is associated with IRF4 expression in B-cell lymphomas, including EBV-associated lymphomas and DLBC (12). The human LIMD1 promoter and the predominant transcription start sites (TSS) were characterized (8), and Pu.1 was reported to be the major transcription factor that activates LIMD1 transcription (56). Of great interest, we identified at least one conserved functional NFκB-binding site and an ETS/ISRE-consensus element (EICE) upstream of the TSS; the latter can bind to the oncogenic transcription factor IRF4 (11). Correspondingly, we showed that LIMD1 is transcriptionally regulated by both IRF4 and NFκB downstream of LMP1 signaling in EBV-transformed cells (11).

Considering its downregulation in NSCLC, LIMD1 was shown to be regulated by HIF1α under hypoxia in a negative feedback loop (4). Interestingly, our results suggest that p53 may transcriptionally activate LIMD1 in LUAD (**Figure 4G**), in addition to a partial contribution from its promoter methylation (**Figures 2F-I**) and from the mutation of specific genes (**Supplementary Figure 5**). Of specific interest, our results show that LIMD1-AS1, a NAT lncRNA of LIMD1, perfectly correlates with LIMD1 at the transcriptional level in various cancers. In general, NAT lncRNAs are able to regulate expression of their sense protein-coding partner genes in *cis* (so called *cis*-NATs) (57–62). In line with our findings, LIMD1-AS1 was recently shown to stabilize LIMD1 mRNA in lung cancer (41). Apart from these epigenetic mechanisms, it is worthy of further investigation of potential transcriptional regulation mediated by other transcription factors, which may be lung cancer specific. In fact, LIMD1 is transcriptionally deregulated in response to various drug treatments that modulate signaling pathways mediated by various receptors, kinases, transcription factors, and others (**Supplementary Table 5**).

LIMD1 has both cytoplasmic and nuclear roles, due to its ability to shuttle between the cytoplasmic and nuclear compartments. It is known to be engaged in the regulation of HIF1α, Wnt and Hippo, and cytoskeletal pathways in the cytoplasmic compartment (5, 13, 14, 50), and positively regulates microRNA (miRNA)-mediated gene silencing (6). As a multifunctional protein, it also inhibits E2F-mediated gene transcription in the nucleus (2). Of importance, our meta-analysis implies novel functions and mechanisms of LIMD1 in NSCLC, highlighted by the findings that it significantly correlates with specific immune infiltrating lymphocytes, especially with neutrophils, in both LUAD and LUSC (**Figure 6**), and with a panel of factors involved in membrane trafficking (**Figure 5**). However, LIMD1 deregulation is only significantly associated with the prognosis of LUAD but not that of LUSC, implying that other LIMD1-mediated functions contribute to the prognosis of NSCLC patients. In fact, the LIMD1 expression levels correlate with the mutation of key tumor suppressors and promoters (**Figure 4G**), and with the deregulation of p53 and CDKN2A pathways (**Figure 8D**), only in LUAD, not in LUSC, suggesting that the deregulation of these pathways specifically favors LUAD development.

LIMD1 is heavily phosphorylated (PhosphoSitePlus.org), such as Y21, Y179, S233, S272, S277, S316, S421, S424, Y527 in humans, with a few sites experimentally validated that render its different functions (33, 63). Cell-cycle-dependent phosphorylation of LIMD1 may play a role in breast cancer (33). Interestingly, our results suggest that phosphorylation of S233 of LIMD1 may be associated with its function in lung cancer (**Figure 2E**). Indeed, different chemotherapeutic treatments result in distinct site-specific LIMD1 phosphorylation, with the highest frequencies at the sites S272 and S277 (**Supplementary Figure 7**), with experimental validation in lung cancer cell lines showing that phosphorylation of these sites in mitosis is required for mitotic progression and LIMD1 tumor-suppressing activity (63). In addition to site-specific phosphorylation, other post-translational modifications (PTMs), including ubiquitination and acetylation, were identified for LIMD1 by high throughput assays, as shown in ProteomicsDB and PhosphoSitePlus portals (not shown).

Besides these mechanisms, disclosing other potential unknown mechanisms underneath context-dependent LIMD1 deregulation and its promo-oncogenic and anti-oncogenic roles in cancers will be our future priority of study. Importantly, our recently published findings have shown that LIMD1 is associated with DNA damage response and protects B lymphoma cells from DNA damage-induced cell death, which represents a novel mechanism accounting for its oncogenic properties in this setting (11, 64). Of great interest, we have also generated a pool of preliminary data showing that LIMD1 is tightly associated with the autophagy program, which counteracts tumorigenesis at early stages while promotes survival and progression of already established cancers, including lung cancer (65–71). In support of its role in autophagy, our results in this study show that LIMD1 correlates with a pool of membrane trafficking regulators (**Figure 5**).

In summary, this meta-analysis indicates that LIMD1, as a tumor suppressor in lung cancer, is significantly downregulated in NSCLC, and could serve as a prognosis marker for LUAD but not for LUSC. This study represents a breath of cancer patient sample databases, and discloses various potential mechanisms underneath the interaction of LIMD1 with NSCLC, for example, site-specific phosphorylation at S233, S272, and S277 of LIMD1 and LIMD1-mediated immune infiltration in this setting. These underlying molecular mechanisms of LIMD1-mediated anti-oncogenesis in NSCLC merit further experimental validation and elaboration, which will be our priority in the following pursuits.

## Data Availability Statement

The original contributions presented in the study are included in the article/**Supplementary Material**. Further inquiries can be directed to the corresponding author.

## Author Contributions

Conceptualization: LW, SN. Data analysis: SN. Funding acquisition: LW, SN. Methodology: LW, MH, SN. Writing and editing: LW, SN. All authors contributed to the article and approved the submitted version.

## Funding

This work was supported by NIH (CA252986 and DE027314) and ASH, and in part by the NIH grant C06RR0306551.

## Conflict of Interest

The authors declare that the research was conducted in the absence of any commercial or financial relationships that could be construed as a potential conflict of interest.
