# Supplementary material for: Algorithm-Based Meta-Analysis Reveals the Mechanistic Interaction of the Tumor Suppressor LIMD1 With Non-Small-Cell Lung Carcinoma
Source: Front Oncol. 2021 Mar 31;11:632638. doi: 10.3389/fonc.2021.632638 (PMC8044451; doi:10.3389/fonc.2021.632638)
Supplement: Supplementary file 1 [file DataSheet_1.pdf]

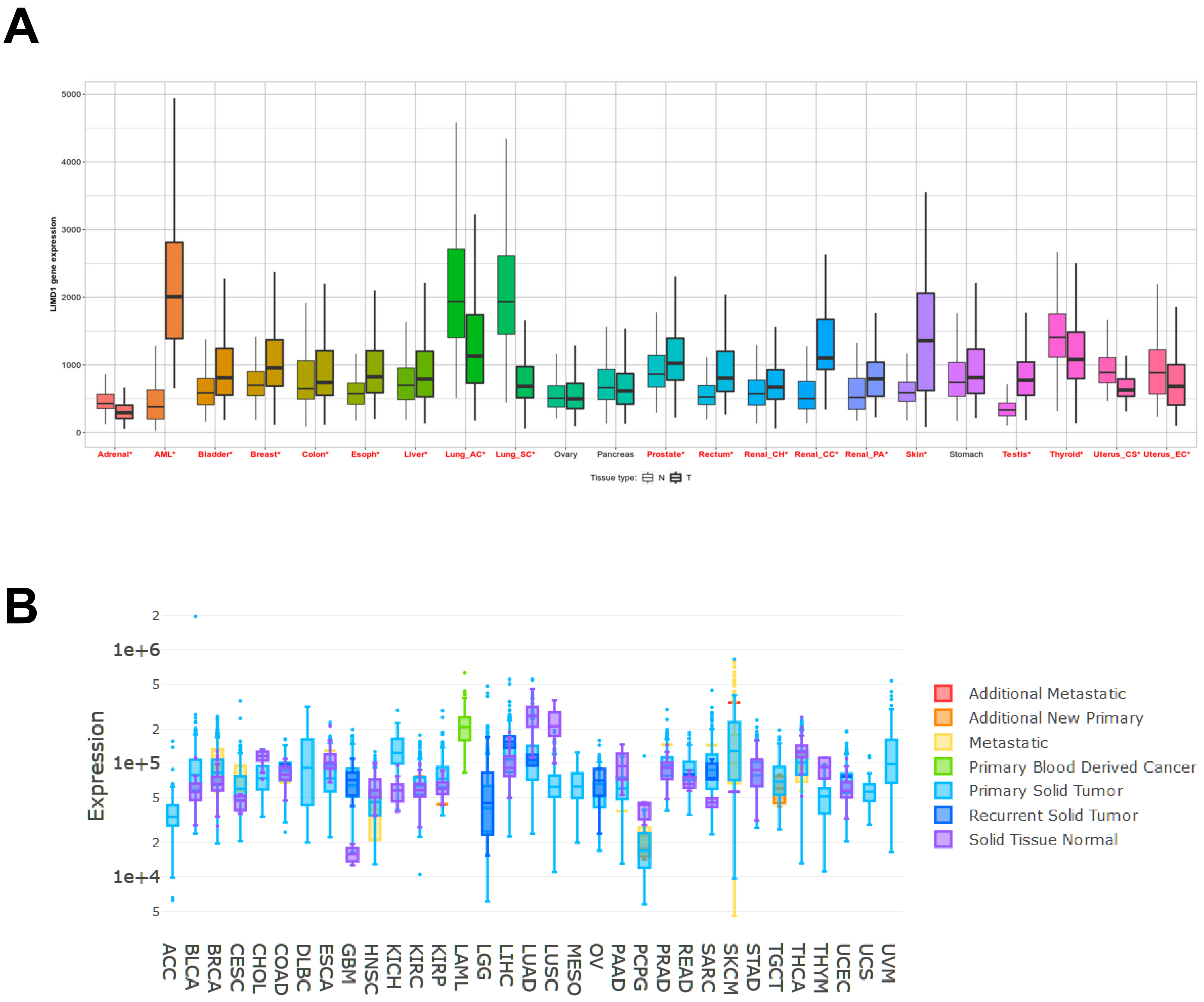

Fig S1. Deregulation of LIMD1 at the transcriptional level in various cancers, as analyzed in TNMPlot (**A**), and DriverDbv3 (**B**)

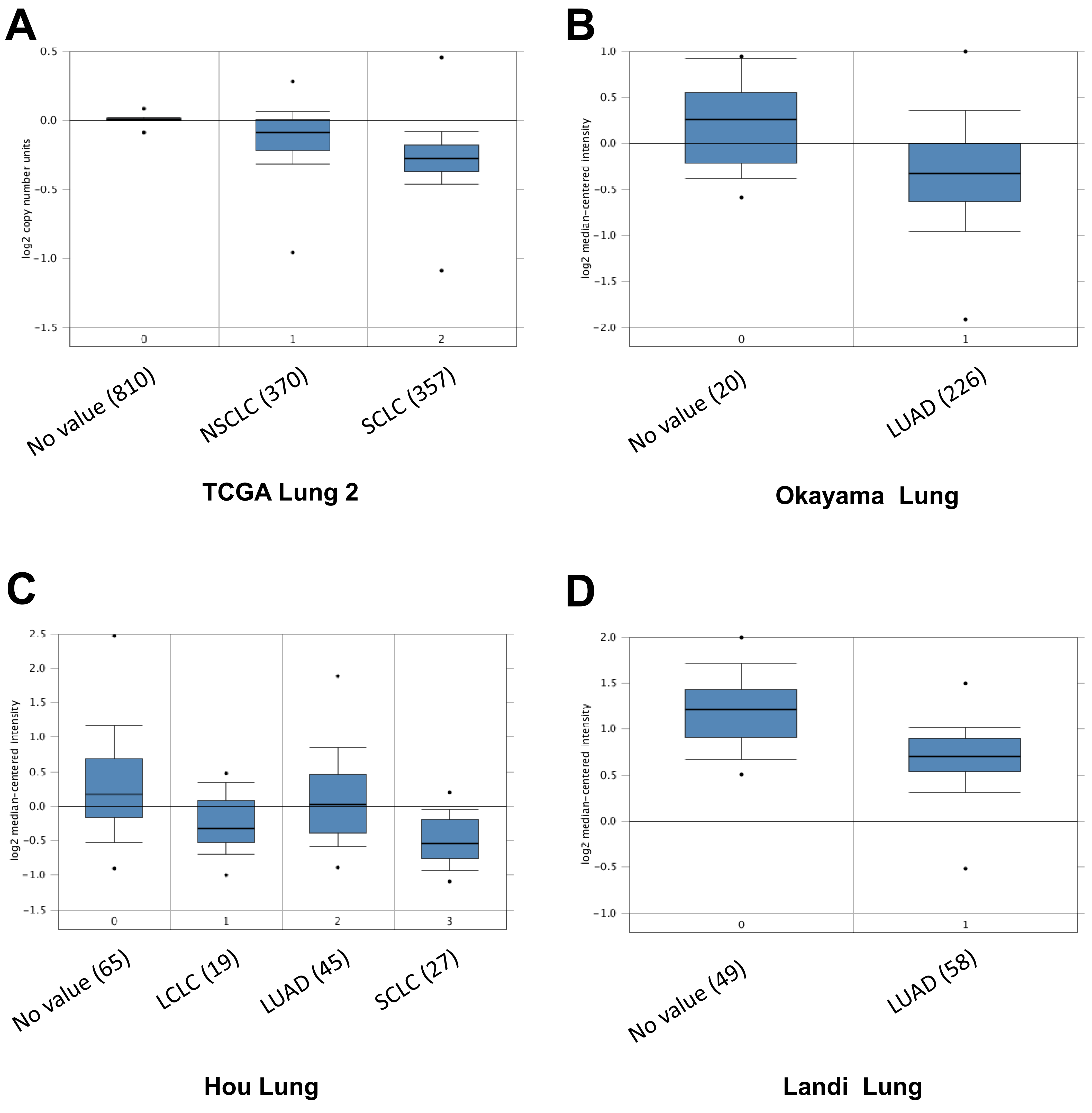

Fig S2. Downregulation of LIMD1 at the transcriptional level in lung cancer, as analyzed with different datasets in Oncomine

Fusion 'LIMD1'-'LARS2' in LUAD

	5'Gene	3'Gene
Gene Name	LIMD1	LARS2
Chromosome	3	3
Strand	1	1
Breakpoint position	45707209	45565489
Discordant read pairs	15	
Junction spanning reads	8	
Perfect Junction spanning reads	6	
BLAST E value	0.045	
Tier	tier1	
Frame	In-frame	
Centrality	0.36997	
SampleId	TCGA.78.7147.01A	
Validated by WGS	NA	

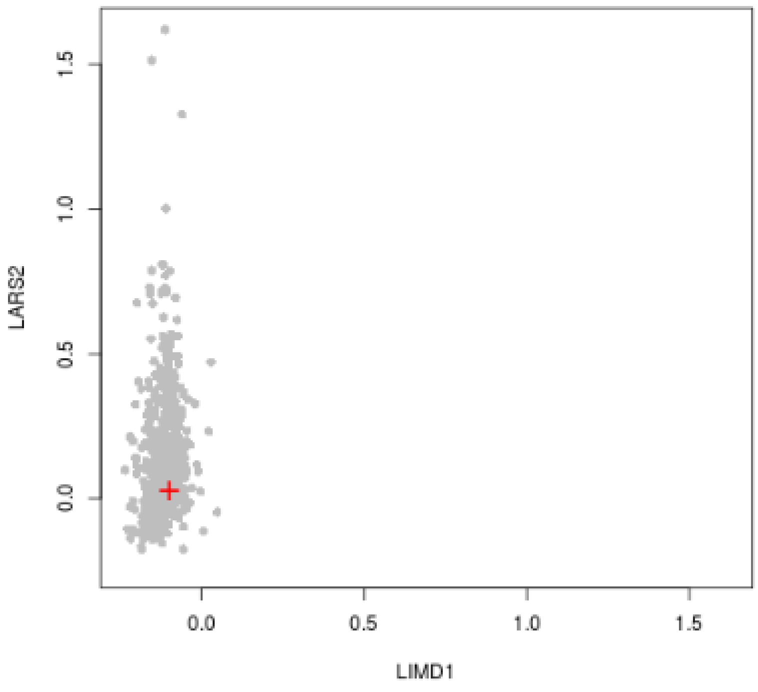

Fig. The gene expression correlation between fused genes

5' Gene	LIM domains containing 1 [Source:HGNC Symbol;Acc:HGNC:6612]
3' Gene	leucyl-tRNA synthetase 2, mitochondrial [Source:HGNC Symbol;Acc:HGNC:17095]

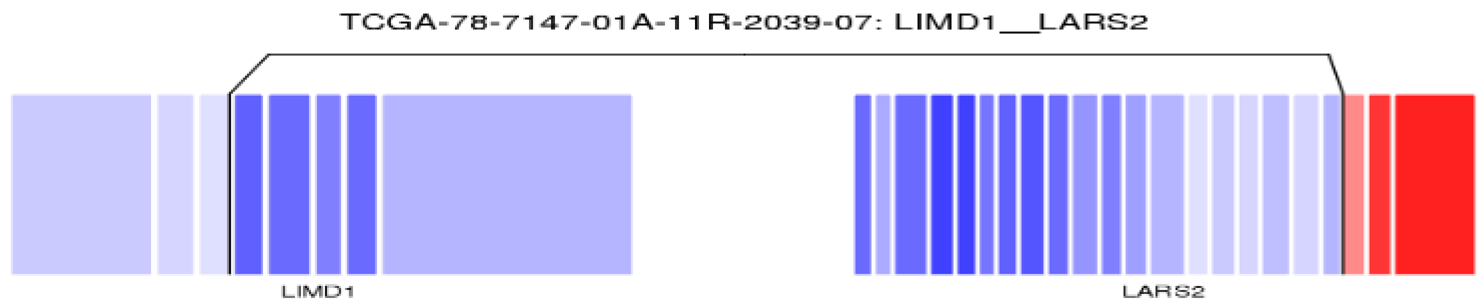

Fig. Exon expression plot for fused genes. Expression was normalized across all exons; blue = lowest expression, red = highest expression. Line indicates where genes are connected.

Fig S3. Diagram of LIMD1-LARS2 fusion details in NSCLC, as illustrated in TumorFusions

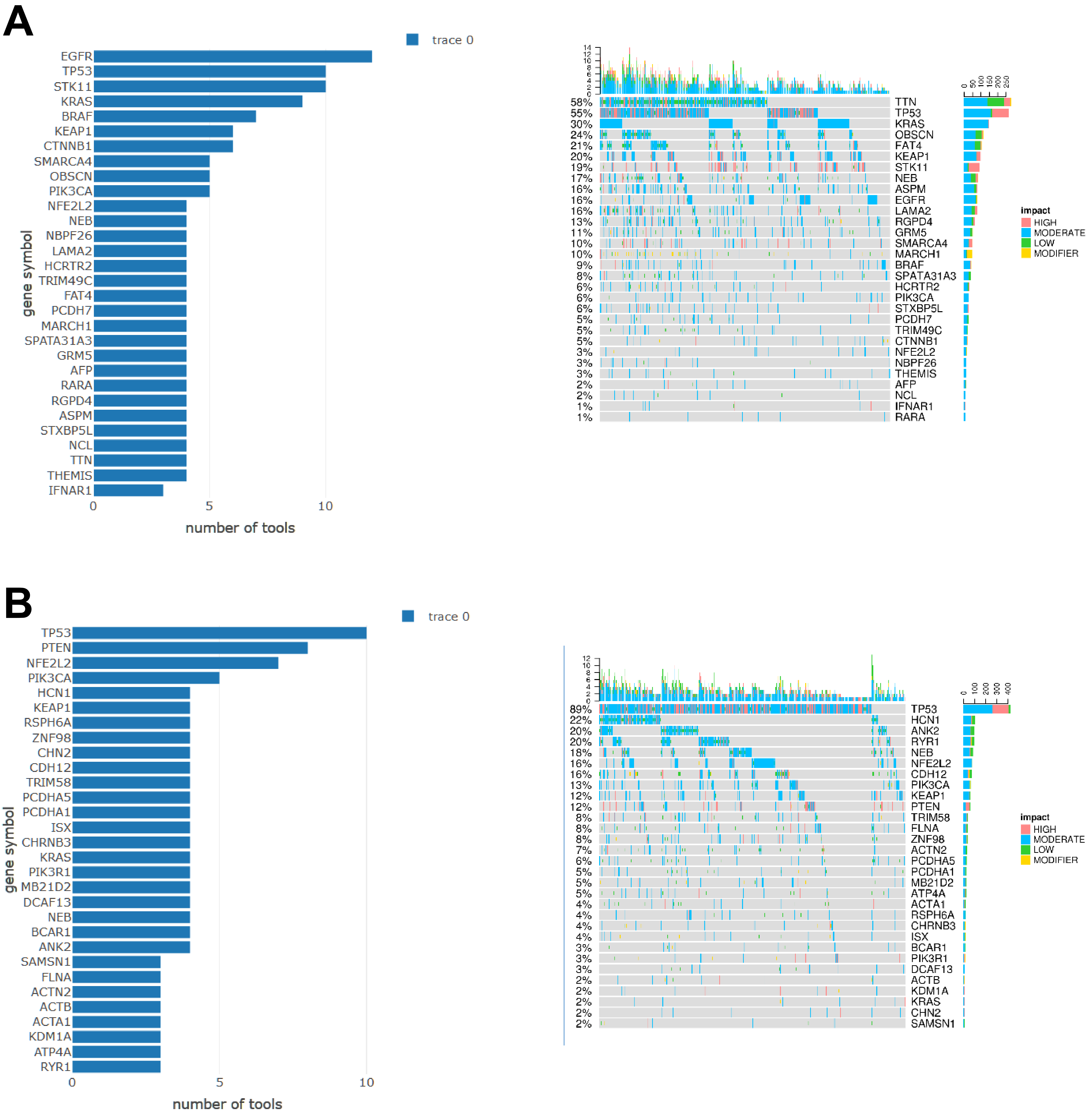

Fig S4. A. Top 30 genes mutated in LUAD and the effect of mutation on their expression; **B**. Top 30 genes mutated in LUSC and the effect of mutation on their expression

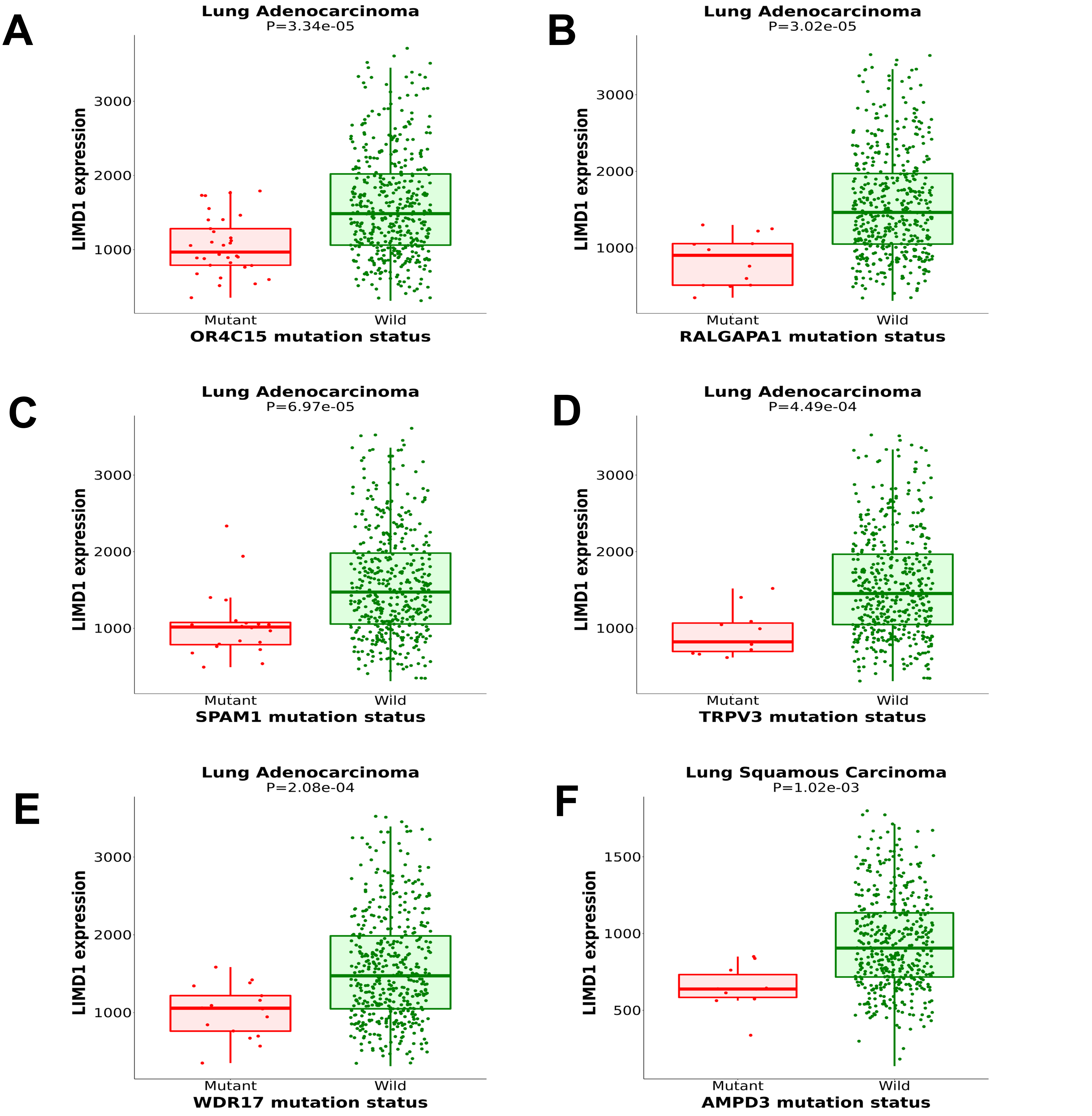

Fig S5. Mutation of top genes in regulating LIMD1 expression in NSCLC. **A-E**. LUAD; **F**: LUSC. Running parameters: Mutation prevalence at least: 2%; Mutation filter: Off; p value cutoff: 0.01; Fold change cutoff: 1.44; FDR cutoff: No FDR filter. LIMD1 expression is show as TPM.

6 search results for **LIMD1**

#	⊕	SuperPathway Name	Genes Count	Relevance Score
1	+	Hippo signaling pathway - multiple species	29	0.741
2	+	Tyrosine Kinases / Adaptors	148	0.371
3	+	Hippo signaling pathway	157	0.371
4	+	Cytoskeletal Signaling	308	0.259
5	+	Cellular Senescence (REACTOME)	452	0.185
6	+	Regulation of activated PAK-2p34 by proteasome mediated degradation	776	0.148

Fig S6. LIMD1-associated pathways, as analyzed in Pathwaycard

Treatment:

anti-CD3	S296-p , S314-p
doxycycline	S272-p , S277-p
EGF	S272-p , S277-p
ischemia	S277-p , T294-p , S316-p , S421-p
lapatinib	S423-p , S424-p
MLN8054	S233-p
nocodazole	S272-p , S277-p , S421-p , S424-p
RO-3306	S272-p , S277-p
SB202190	S272-p , S277-p
selumetinib	S272-p , S277-p
siRNA	S272-p , S277-p
SP600125	S272-p , S277-p
Su11274	Y179-p
taxol	S272-p , S277-p
thymidine	S272-p , S277-p
vemurafenib	S272-p , S277-p

Fig S7. Site-specific phosphorylation of LIMD1 in response to various drug treatments, as analyzed in PhosphositePlus
